# Mid-Term Outcomes, Biological Responses and Complications of Dental Implants in Maxillomandibular Reconstruction with Free Bone Flaps: A Systematic Review and Meta-Analysis

**DOI:** 10.3390/diagnostics16030435

**Published:** 2026-02-01

**Authors:** Siqi Qiu, Kuo Feng Hung, Feng Wang

**Affiliations:** 1 Oral and Maxillofacial Surgery, Faculty of Dentistry, The University of Hong Kong, Hong Kong SAR, China; qiu-siqi02@connect.hku.hk; 2 Oral and Maxillofacial Radiology, Applied Oral Sciences and Community Dental Care, Faculty of Dentistry, The University of Hong Kong, Hong Kong SAR, China; hungkfg@hku.hk

**Keywords:** mandibular reconstruction, maxillary reconstruction, dental implants, osteocutaneous flaps, implant success

## Abstract

**Background/Objectives:** Maxillofacial reconstruction with a vascularized free bone flap for facial contour restoration serves as a foundation for dentition rehabilitation. Although state-of-the-art studies have reported promising results with implant-supported prostheses in such cases, evidence for dental implant prognosis remains insufficient. This study aims to synthesize the mid-term clinical outcomes of implants placed in vascularized free bone flaps, taking into account the biological responses and associated complications. **Methods:** Studies with a minimal 3-year follow-up, no less than 10 patients, and reporting implant survival/success rate were included. Literature published from 2000 to 2025 was collected from PubMed, Embase, and Scopus. Meta-analyses were performed to pool the implant survival and success rates for the entire cohort, the biological complication rates, the odds ratio for radiotherapy, and the pooled implant failure rates associated with radiotherapy. Parameters related to biological prognosis were collected. ROBINS-E and NOS scale were used to assess the risk of bias. **Results:** Of the 949 records identified, 14 retrospective and 2 cohort studies were included, yielding a total of 1165 dental implants placed in free bone flaps. On the implant level, meta-analysis demonstrated a pooled implant survival rate of 97.9% (95% CI: 0.922–0.994, *I*^2^ = 64.4%) and a pooled implant success rate of 88.1% (95% CI: 0.803–0.931, *I*^2^ = 68.3%). The pooled biological complication rate was 8.6% (95% CI: 0.052–0.138; *I*^2^ = 69.5%). Among patients who underwent radiotherapy, the pooled implant failure rate was 13.7% (95% CI: 0.087–0.210; *I*^2^ = 0.0%; *p* = 0.4702) with an odds ratio of 3.086 (*I*^2^ = 66.5%) for radiotherapy-associated implant failure. **Conclusions:** Implant-related outcomes in these complex cases are generally acceptable, with high survival, moderately high success rates and overall stable biological response. Additionally, radiotherapy adds to the risk of implant failure on implant level. However, the statistical heterogeneity and inconsistent definitions of biological outcomes in the literature suggest that caution is warranted when planning implant therapy in these cases. Further studies with long-term follow-up, focused on peri-implant tissue conditions and adopting more stratified study designs to minimize confounding factors, are needed.

## 1. Introduction

Over the past decades, reconstruction of the jaw with vascularized free bone flaps has become a well-established and widely accepted procedure for restoring maxillomandibular continuity following severe trauma, cysts, or ablative tumor surgery [1,2,3]. This reconstruction with an osteocutaneous flap could partially retrieve masticatory and speech function. At the same time, increasing attention has been focused on a more complete restoration, including dentition, which has been considered essential for aesthetic and functional recovery [4,5,6,7]. Rehabilitation with conventional prostheses is often unsuitable and impractical in reconstructed jaws because the reconstructed soft and hard tissues present challenges for prosthesis retention and are less resilient to masticatory forces, which may lead to patient-reported discomfort [8,9,10]. Additionally, considering the stability and security of endosteal implantation, rehabilitation after implantation has been a feasible option for patients who underwent maxillomandibular reconstruction with free bone flaps [11,12]. However, the distinct embryologic origins of the native alveolar bone, derived from the neural crest ectomesenchyme and the grafted free bone flap, coupled with the complex soft tissue environment, contribute to the significant challenges associated with this procedure [13,14,15]. In addition, the potential need for radiation therapy in patients is a major concern for invasive operation [13,14,16,17,18].

A substantial number of studies have reported implant survival and success after maxillomandibular reconstruction with vascularized free bone flaps in diverse patient populations, encompassing variation in flap type, implant timing and exposure to radiotherapy [19,20,21,22,23,24]. However, most available data arise from small, single-center retrospective case series, with limited control of confounding and variable follow-up, which reduces the overall certainty of the evidence. Existing reports have largely focused on technical and short-term surgical outcomes, whereas long-term peri-implant tissue health, biological complications and functional prognosis have been described only sporadically. In addition, the criteria used to define “implant success” are inconsistent and often incompletely reported across studies [25], hindering comparisons between cohorts and risking overly optimistic interpretation in clinical practice. These limitations highlight the need for a systematic synthesis that not only summarizes implant survival and success but also critically examines peri-implant health.

This study aims to summarize the mid-term clinical outcomes of implants placed in vascularized free bone flaps, taking into account the biological responses and associated complications. The primary objective is to perform a systematic review and meta-analysis of the survival and success rates of dental implants placed in vascularized free bone flaps. The secondary objective was to pool the complication rate, the failure rate of implants receiving radiotherapy, the risk of radiotherapy to implant failure and also discuss and summarize the biological responses after implantation in clinical practice.

## 2. Materials and Methods

A comprehensive electronic literature review was conducted according to the guidelines of the PRISMA statement (Preferred Reporting Items for Systematic Reviews and Meta-Analysis) [26]. This systematic review was registered in the International Prospective Register of Systematic Reviews (PROSPERO) with the identification number (CRD420251125706). While the primary outcomes and overall methodology remained consistent with the registered protocol, minor modifications were made to the risk of bias assessment. These modifications were implemented prior to data extraction and analysis to ensure methodological rigor and transparency.

### 2.1. Search Strategy and Inclusion Criteria

An extensive literature search was conducted on 15 August 2025. Relevant studies published from 2000 to 2025 were identified through the PubMed, Embase, and Scopus databases. Terms for maxillary and mandibular reconstruction with a vascularized flap and dental implantation were used as defined in the database. MeSH terms, Emtree terms and free-text keywords were combined, and Boolean operators and truncation were used. The search strategy is given in the Supplementary Document.

**Figure 1:**
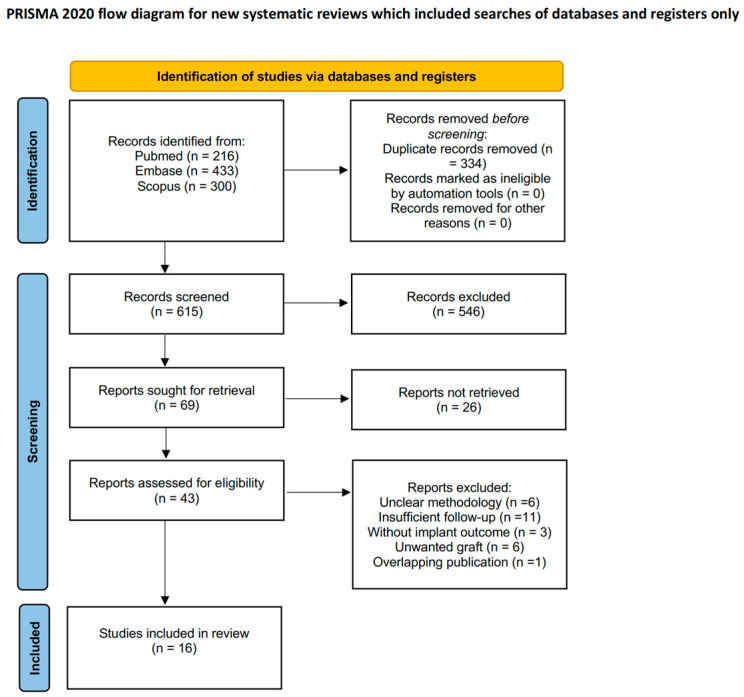
PRISMA flow chart. Source: Page, M.J., et al. *BMJ* **2021**, *372*, n71. https://doi.org/10.1136/bmj.n71 [26]. This work is licensed under CC BY 4.0. To view a copy of this license, visit https://creativecommons.org/licenses/by/4.0/. Accessed on 17 August 2025.

Inclusion criteria include (1) articles published in English, (2) original retrospective/prospective studies involving patients receiving dental implantation after maxillofacial reconstruction with free bone flaps, and (3) studies reporting a minimum average follow-up of 3 years. For studies reporting multiple time points, we selected the time point ≥3 years for data extraction and meta-analysis. The exclusion criteria include (1) reviews, posters, abstracts, and animal studies; (2) studies without reporting implant outcomes in reconstructed bone separately from implants placed in native bone or machined surface implant (“unclear methodology” in Figure 1); (3) studies with fewer than 10 patients; (4) studies with insufficient information on implant survival/success rate; (5) studies with insufficient follow-up; (6) studies with overlapping data; and (7) studies with unwanted design using non-vascularized transplant (“unwanted graft” in Figure 1).

Figure 1 displays the PRISMA flowchart for the study selection process. Results of the search were imported into EndNote 21.5 literature manager software. Duplicates were automatically excluded by EndNote and manually checked by investigators; undetected records were screened manually in a subsequent publication screening process using titles and abstracts. The specific reasons for exclusion after screeding were as follows: unclear methodology (*n* = 6), insufficient follow-up (*n* = 11), without implant outcome (*n* = 3), unwanted graft (*n* = 6), and overlapping publication (*n* = 1). Full-text articles were screened independently by two investigators (SQ and KFH). In the event of any disagreement between the two investigators, a third investigator (FW) was introduced into the process.

### 2.2. Data Collection

Data were extracted using a structured data collection form in Excel spreadsheets by two independent investigators (SQ and KFH). All discrepancies were resolved through discussion, and the authors were contacted when further information was required. Demographics and clinical information (author’s last name, year of publication, type of study, age, gender, histopathology, defect location, data on adjuvant radiotherapy, and follow-up period) were manually extracted and summarized. Data on implant-related variables were collected, including sample size, implant placement timing and implant status (failed/slept and surviving/successful within the free bone flap). The “slept” implant here indicates a condition where the implant is no longer in function but is still present and osseointegrated. Additionally, peri-implant parameters—such as marginal bone loss, probing depth (PD) and bleeding on probing index—as well as the use of soft tissue grafts, biological complications and their management were recorded. No assumption was made when data were missing.

The primary outcome was implant survival/success at the most recent available follow-up in the overall cohort of selected studies. The secondary outcome was the risk of radiotherapy for implantation in free bone flaps or implant failure in patients who underwent radiotherapy. Implant-related clinical outcomes (i.e., peri-implant marginal bone loss, PD, bleeding index, implant-related biological complications and effect of soft tissue graft) were noted. Success of the implant was defined according to Albrektsson criteria [27]: (a) painless, (b) no question, (c) no mobility and (d) no peri-implant radiolucency and peri-implant resorption less than 1.5 mm at the first year of function, and 0.2 mm in the subsequent years, or van Steenbergher criteria [28]: absence of implant-related pain, suppuration, swelling, mobility, discomfort, ongoing pathological processes, peri-implantitis, neuropathies, or persistent paresthesia. Implant success rate was only included in the final assessment if it was clearly stated by the authors.

### 2.3. Quality Assessment

Two investigators (SQ and KFH) conducted a quality assessment of the included studies. Risk Of Bias In Non-randomized Studies-of Exposures (ROBINS-E) was used to assess retrospective studies, case series and case reports, since there is no appropriate scale for these assessments [29]. Quality was described as low, some concerns, high and very high. The Newcastle–Ottawa Scale was used to assess risk of bias in nonrandomized cohort studies. A study with a final score of 0 to 2 was considered high risk of bias, moderate when a score was 3 to 5, and low when a score was 6 to 9. Certainty in the body of evidence for each outcome was assessed using a GRADE-inspired framework, considering study limitations (risk of bias), inconsistency, indirectness, imprecision and potential reporting bias. Overall certainty was rated as high, moderate, low, or very low.

### 2.4. Statistical Analysis

The quantitative analysis was performed in R version 4.2.0 (R Foundation for Statistical Computing, Vienna, Austria) using the meta package (version 6.5-0). Not all included studies contributed data to every synthesis. All studies were counted in the overall description of implant numbers, but a study was entered into a given meta-analysis only if it reported that specific outcome with an explicit definition and sufficient numerical data. Consequently, some series that reported overall implant survival but did not provide separate data or clear criteria for implant “success”, complications or radiotherapy-related failures contributed to the survival analysis only and were excluded from the corresponding meta-analyses.

Study-specific effect estimates with 95% confidence intervals (CIs) were first calculated and then combined using an inverse-variance random-effects meta-analysis using logit transformation with leave-one-out sensitivity analysis to assess the robustness of the pooled results. The pooled results were presented as forest plots, with confidence intervals and weights displayed. Statistical significance was defined as a *p*-value < 0.05. Risk of bias due to missing results (reporting biases) was assessed at the level of each synthesis. For outcomes with at least ten contributing studies, funnel plots of effect size against standard error were used to inspect asymmetry visually, and Egger’s regression test for small-study effects was applied where appropriate. Egger’s linear regression test for funnel plot asymmetry was applied only to syntheses including at least 10 studies; for outcomes with fewer than 10 studies, funnel plots were inspected qualitatively and no regression-based tests were performed because of low power and poor interpretability. In these cases, funnel plots were qualitatively inspected, and the risk of reporting bias was assessed based on the size and design of contributing studies, selective outcome reporting and the plausibility of unpublished negative or null results.

## 3. Results

### 3.1. Study Selection

Of the 949 records identified, 16 studies were included based on predefined inclusion and exclusion criteria (Figure 1) [4,8,14,30,31,32,33,34,35,36,37,38,39,40,41,42]. Studies included two prospective studies [30,37] and 14 retrospective cohorts [4,8,14,31,32,33,34,35,36,38,39,40,41,42] (Table 1).

### 3.2. Patients’ Characteristics

Patient characteristics are presented in Table 1. Among the 16 studies, a total of 388 patients (137 females and 223 males) underwent vascularized free bone flap reconstruction and primary or secondary implantation, with an average age of 50.3 years (range, 12–80). As Iizuka et al. reported multiple oral rehabilitation methods, implant-treated patients’ demographic characteristics were not accessible [4]. The etiology for maxillomandibular osteotomy was reported in a mixed manner, except for two studies [34,39] reporting ameloblastoma only. Within accessible information, 118 patients were diagnosed with squamous cell carcinoma, 78 patients were reported with ameloblastoma and other diagnoses included keratocyst, trauma, osteomyelitis, atrophy, osteoradionecrosis, agenesis and micrognathia.

Fifteen studies [4,8,14,30,31,32,33,34,35,37,38,39,40,41,42] with 288 patients used the free fibula flap for reconstruction, while three studies [8,36,37] with 36 patients included iliac crest grafts, and one study [8] with 3 patients received radial artery free flaps. The start time of follow-up varied in each study, with four studies starting at staged implantation [4,8,36,38], seven studies starting at prosthesis delivery [30,34,37,39,40,41,42] (one emphasized functional rehabilitation [39]), one study starting at ablative surgery [14], and four studies without mentioning the start time [31,32,33,35]. Since Wiesli et al. [14] reported a long-term follow-up, we included this study, although its follow-up began at the time of ablative surgery. The median follow-up time was 44.5 months, with a minimum follow-up of at least 3 years.

### 3.3. Implant-Related Outcomes

A total of 1165 implants in free bone flaps from 388 patients was reported (Table 2). Of these, a total of 716 implants, which included 221 patients with 596 implants explicitly reported, underwent delayed implantation after reconstruction. The interval between reconstruction and implantation was not standardized, ranging from 4 to 38 months. The interval time for implantation was differentiated among different reconstructed mandibular augmentation techniques, namely vertical distraction, iliac bone graft, and double-barreled fibula, as given in Table 2 [30,31,41]. In addition to secondary implantation, 174 implants were placed in free bone flaps during flap transplantation, with 26 patients receiving 109 implants [4,30,32,34,39]. Two studies did not report exact patient or implant counts stratified by implant timing [32,40]. Two studies [37,42] did not report the interval time for implantation, though patients received a secondary implantation.

In the available records, radiotherapy was administered to 111 patients [4,8,31,32,33,35,37,38,40,42], with 98 patients receiving radiotherapy before implantation and 13 patients receiving radiotherapy after implantation. These patients received 137 implants (five of which received radiotherapy after implantation), with no information on the number of implants in certain studies [32,33]. Among patients who received radiotherapy prior to implantation, except for certain studies [32,33,42], the interval between radiotherapy and implantation was at least 12 months (Table 2). The rate of failed implants with radiotherapy was reported in [30,31,35,37,38,40,42], with one study reporting postoperative radiotherapy [42]. Implant failure was defined as implants that are extracted or slept, with the latter indicating no functions.

Except for free bone flaps receiving vertical distraction, all the fibula flaps included in the studies had a skin paddle. Herein, soft tissue management was reported in several studies, including palatal mucosa graft, debulking of flap tissue followed by free mucosal grafts, sub-periosteal dissection with denture guided epithelial regeneration, connective tissue and skin graft, collagen matrix and thinning skin paddle [4,8,30,34,35,37,38,40,42], among which palatal mucosa graft was the most used for improved keratinized mucosa condition.

Survival of the implant was defined as the absence of peri-implant bone loss/no implant loss, with loaded implants in function and no mobility, pain, or infection. Survival rates ranged from 73.91% to 100%. It is noteworthy that the result reported by Shaw et al., which showed the lowest implant survival (73.9%), differed substantially from the other included studies. This series combined multiple types of free bone flaps and included a large proportion of patients reconstructed for squamous cell carcinoma. These factors may partly explain the lower survival rate observed in that study. The success criteria were given before literature screening, the included studies for success rate analysis were using the Albrektsson criteria, and the rate ranged from 73.50% to 100%.

Jaw-in-a-day technique (JIAD) [43], involving single-stage reconstruction with simultaneous implant placement and immediate prosthetic rehabilitation, was not explicitly described in the included series, many of which predate the widespread adoption of this technique. Therefore, the present findings mainly reflect staged implant rehabilitation rather than contemporary JIAD protocols. For the prosthesis delivery time, the waiting duration varied from 4 months to 15 months, with four studies extending the interval time of prosthesis delivery to more than 6 months [34,37,39,42] and three studies sticking to the conventional 4–6 months [31,38,40], with two shorter reports [30,41].

Table 3 demonstrates the prognosis of implants. Among the included studies reporting peri-implant parameters, probing depth (PD) and bleeding index were less frequently reported than marginal bone loss. Reporting formats were highly heterogeneous; we did not perform a quantitative meta-analysis for marginal bone loss and instead summarized the available data narratively. Marginal bone loss was detected in nine studies [14,30,31,33,34,38,39,41,42], with an average bone loss reported in five studies [30,31,38,39,41] and most marginal bone loss less than 2 mm [14,30,31,33,34,39]. Other studies presented marginal bone loss outcomes with a range; one showed that MBL was less than 1.5 mm [34], and the others showed that around three-quarters of implants had an MBL ranging from 0.6 to 1.5 mm [42] or the largest portion had an MBL of less than 1 mm [33]. Additionally, two studies reported the yearly change in bone loss [41,42]. MBL parameters were obtained with panoramic radiographs, and cone-beam computed tomographic (CBCT) scans [30] and periapical x-ray [39]. PD of implants could be obtained in five studies [33,38,39,42]; the range of PD achieved the most outcomes with less than 4 mm. A deep probing depth exceeding 4 mm was also reported in a study [33], with acceptable cortical bone loss and stable implant mobility. Two studies reported MBL and PD at annual follow-up [41,42]. Bleeding index was reported in four studies [14,33,41,42], with two reporting the modified sulcus bleeding index (mSBI) [33,41,42] and one reporting the papillary bleeding index (PBI) [14]. The bleeding index could not be pooled because the criteria were inconsistent. One study reported mSBI using the Muhlemann–Son scale based on soft tissue appearance, bleeding on probing, color changes and edema, and two other studies [41,42] defined mSBI using the same scale. The other one used the papillary bleeding index (PBI) defined by Saxer and Mühlemann.

Implant-related complications included lack of osseointegration at the early stage [32,33,36], peri-implantitis [14,27,32,33,35,37,38,40], peri-implant mucositis [38], overgrowth of granulomatous tissue [38,41,42], late failure [33,37] and other implant-related failures. The complication rate ranged from 1.73% to 25%.

### 3.4. Meta-Analysis

Single-arm proportional meta-analyses were used to calculate pooled implant survival/success rate, complication rate and implant failure rate with radiotherapy.

**Figure 2:**
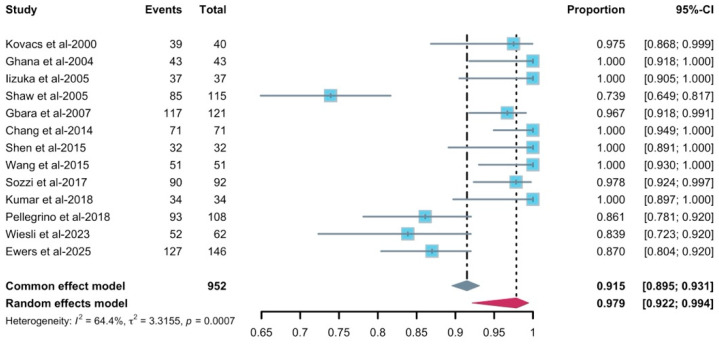
Forest plot of implant survival rate [4,8,14,30,32,33,34,36,37,38,39,40,41]. The gray and purple symbol demonstrate the pooled proportion and its 95%-CI.

**Figure 3:**
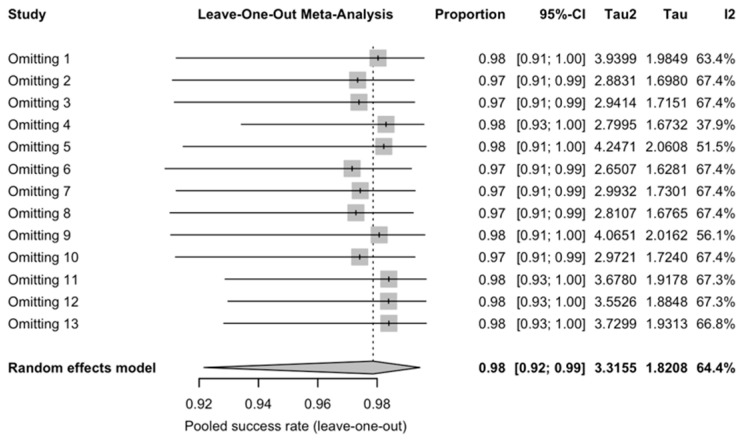
Forest plot of leave-one-out meta-analysis for implant survival rate. 1. Kovacs et al. [36], 2. Ghana et al. [34], 3. Iizuka et al. [4], 4. Shaw et al. [8], 5. Gbara et al. [33], 6. Chang et al. [30], 7. Shen et al. [39], 8. Wang et al. [41], 9. Sozzi et al. [40], 10. Kumar et al. [37], 11. Pellegrino et al. [38], 12. Wiesli et al. [14], 13. Ewers et al. [32]. The rhombus symbol demonstrates the pooled proportion and its 95%-CI.

**Figure 4:**
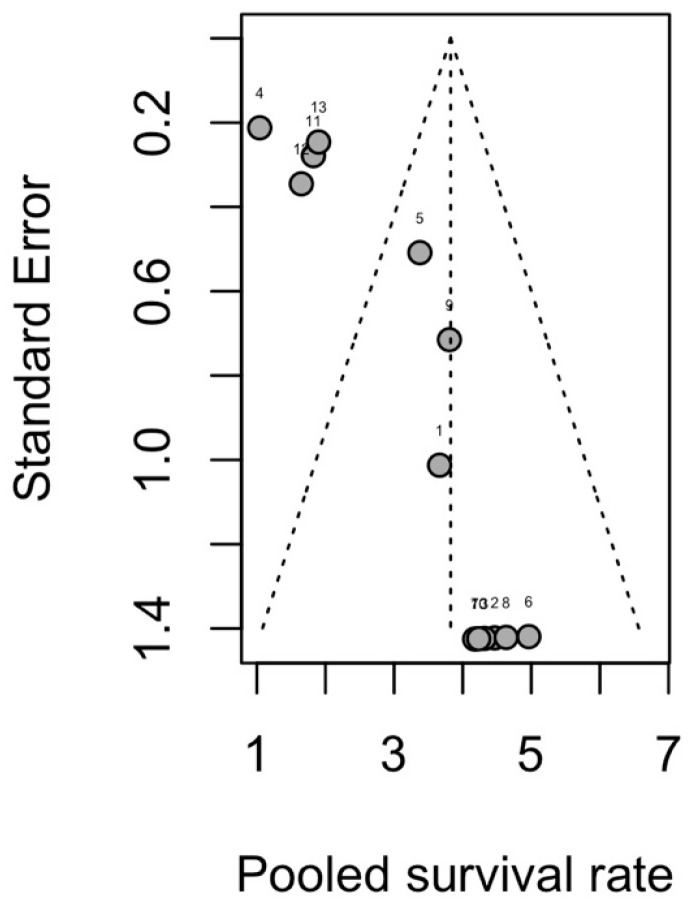
Funnel plot for implant survival rate. 1. Kovacs et al. [36], 2. Ghana et al. [34], 3. Iizuka et al. [4], 4. Shaw et al. [8], 5. Gbara et al. [33], 6. Chang et al. [30], 7. Shen et al. [39], 8. Wang et al. [41], 9. Sozzi et al. [40], 10. Kumar et al. [37], 11. Pellegrino et al. [38], 12. Wiesli et al. [14], 13. Ewers et al. [32].

A pooled analysis of 12 studies that included 952 implants placed in free bone flap revealed a total implant survival rate (97.9%, 95–CI: 0.922–0.994, Figure 2). Heterogeneity was substantial (*I*^2^ = 64.4%, *p* = 0.0007). Leave-one-out influence analysis (Figure 3) showed that omission of any single study did not materially change the pooled survival estimate. All leave-one-out pooled values remained within the 95% CI of the primary random-effects analysis, though *I*^2^ dropped when omitting Shaw et al.’s study, showing moderate heterogeneity. The funnel plot (Figure 4) suggested a notable asymmetry, with several small studies reporting high survival clustered to the right. Egger’s linear regression test confirmed the presence of small-study effects (t = 5.86, df = 11, *p* = 0.0001), indicating a high risk of reporting or publication bias. Subgroup analysis did not reveal clear effect modifiers. When stratified by follow-up duration (60 months), no statistically significant subgroup difference (χ^2^ for subgroup differences *p* = 0.18, (Figure S1) was found. Similarly, no statistically significant subgroup difference was detected for radiotherapy and implant timing (*p* = 0.4352 and *p* = 0.0926, separately).

**Figure 5:**
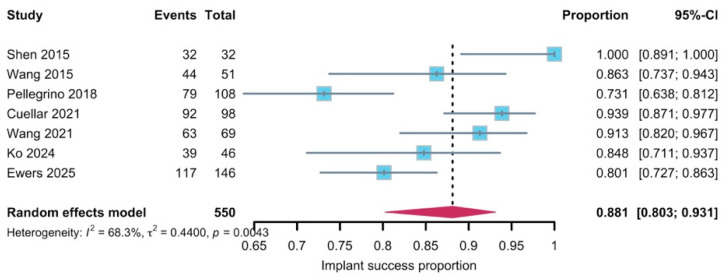
Forest plot of implant success rate [31,32,35,38,39,41,42]. The rhombus symbols demonstrate the pooled proportion and its 95%-CI.

**Figure 6:**
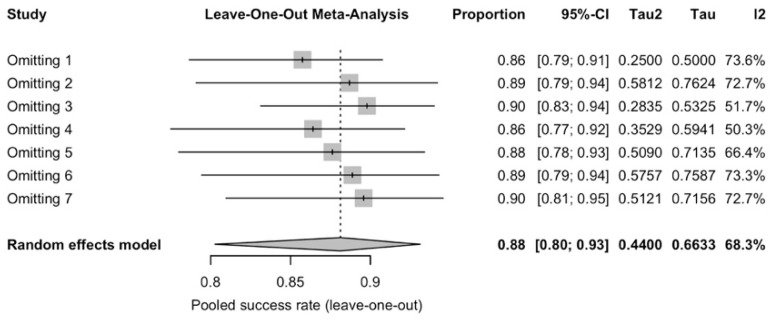
Leave-one-out meta-analysis for implant success rate. 1. Shen-2015 [39], 2. Wang-2015 [41], 3. Sozzi-2017 [40], 4. Pellegrino-2018 [38], 5. Wang-2021 [42], 6. Ko-2024 [35], 7. Ewers-2025 [32]. The rhombus symbol demonstrates the pooled proportion and its 95%-CI.

**Figure 7:**
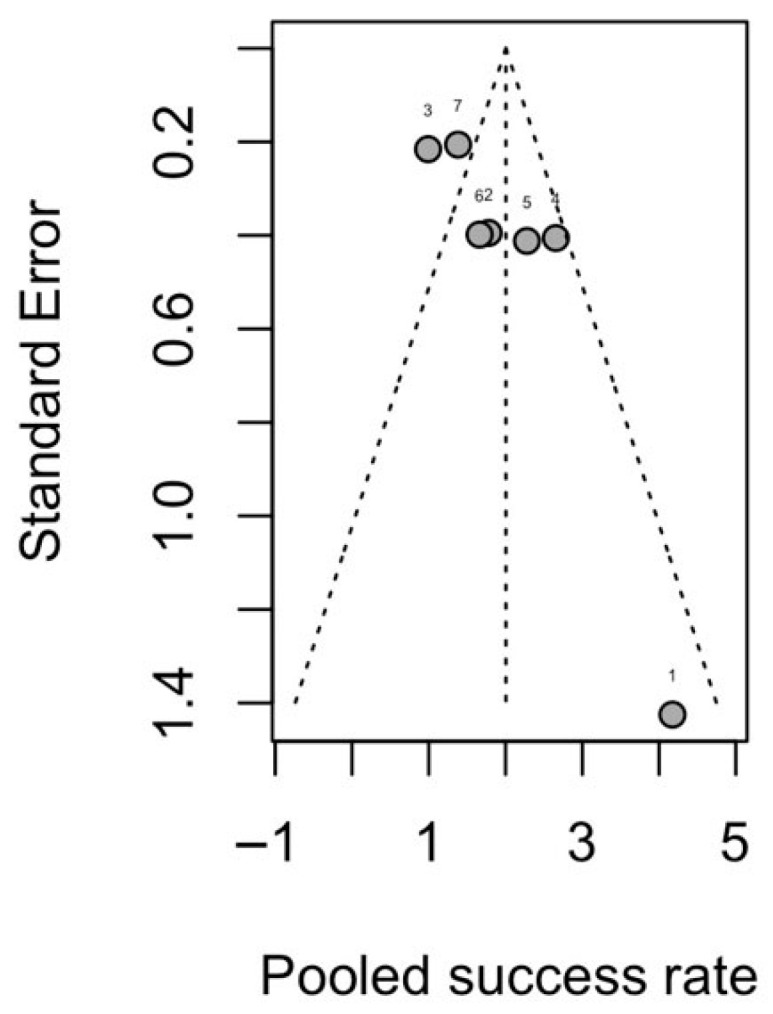
Funnel plot for implant success rate. 1. Shen-2015 [39], 2. Wang-2015 [41], 3. Sozzi-2017 [40], 4. Pellegrino-2018 [38], 5. Wang-2021 [42], 6. Ko-2024 [35], 7. Ewers-2025 [32].

Implant success rate pooled analysis (88.1%, 95–CI: 0.803–0.931, Figure 5) included 550 implants with seven studies, with a substantial heterogeneity (*I*^2^ = 68.3%, *p* = 0.0043) and all reporting with Albrektsson criteria. Leave-one-out influence analysis showed that all pooled values remained within the 95% CI of the primary random-effect analysis (Figure 6). The funnel plot for success showed some visual asymmetry, with smaller studies tending to report more extreme success rates, but interpretation was limited by the small number of studies (Figure 7). Egger’s linear regression test was not applicable in this synthesis, and the risk of reporting bias for the success synthesis should be considered as at least “uncertain”. Subgroup analysis (Figure S2) did reveal that radiotherapy could be a convincing effect modifier (*p* < 0.05), while implant timing did not show a statistically significant subgroup difference (*p* = 0.4428). Subgroup analysis for follow-up was not applicable due to the limited amount of studies.

**Figure 8:**
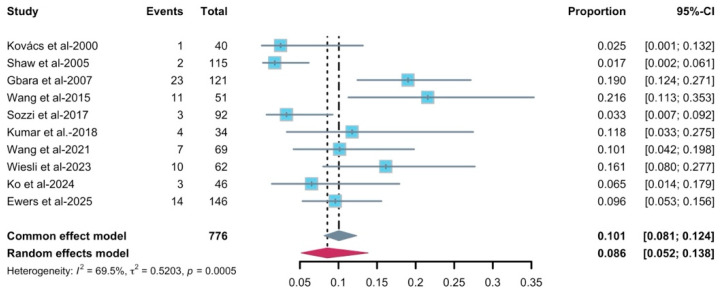
Forest plot of complication rate [8,14,32,33,35,36,37,40,41,42]. The gray and purple symbol demonstrate the pooled proportion and its 95%-CI.

The pooled analysis of complication rate (Figure 8) was 8.6% (95–CI: 0.052–0.138), with a substantial heterogeneity (*I*^2^ = 69.5%, *p* = 0.0005). Nine studies with 776 implants contributed to the meta-analysis. The funnel plot (Figure S3) for complication rate showed some scatter with a tendency for smaller studies to report more extreme proportions, but with only nine studies the pattern was difficult to interpret. In view of the small number of contributing studies (k < 10) and current recommendations, we did not perform Egger’s regression test or other formal tests for funnel-plot asymmetry for this synthesis; therefore, the risk of reporting bias remains uncertain, and the pooled complication estimate should be interpreted cautiously. Leave-one-out influence analysis was conducted for robustness. Omitting each study in turn produced pooled estimates that remained in a similar range, and all fell within the 95% CI of the main random-effects model (Figure S3). Moreover, subgroup analyses were not feasible for this outcome because most studies did not provide complication data stratified by relevant covariates.

**Figure 9:**
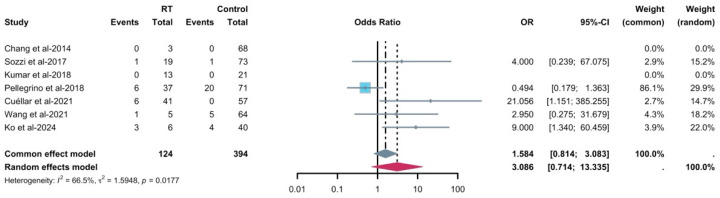
Forest plot of the odds ratio of radiotherapy for implant failure [30,31,35,37,38,40,42]. The gray and purple symbol demonstrate the pooled proportion and its 95%-CI.

Radiotherapy significantly increased the risk of implant failure as well (OR = 3.086, *p* = 0.0177, *I*^2^ = 66.5%), with seven studies including 124 irradiated implants and 394 control implants (Figure 9). For the comparative radiotherapy outcome, because most studies reported radiotherapy exposure and failures as implant-level counts without patient-level cross-tabulations, implants were treated as the unit of analysis, and we were unable to formally adjust for clustering of multiple implants within the same patient. Therefore, the precision of the pooled odds ratio may be somewhat overestimated and the findings should be interpreted cautiously. The funnel plot of OR against standard error (Figure S4) showed some asymmetry, with smaller studies tending to report more extreme effect estimates, but interpretation was constrained by the small number of available studies and low event counts. In line with current recommendations, we therefore did not perform Egger’s regression or other formal tests for small-study effects for this outcome. Leave-one-out analysis was conducted, suggesting that omitting Pellegrino et al. changed the magnitude of the pooled OR but did not qualitatively alter the conclusion that the confidence intervals were wide and overlapped the null value (Figure S4). Subgroup analysis was not feasible in this case.

**Figure 10:**
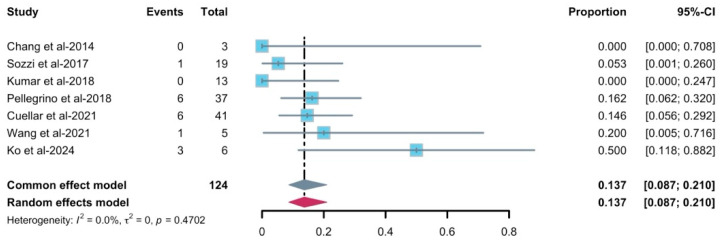
Forest plot of implant failure rate with radiotherapy [30,31,35,37,38,40,42]. The gray and purple symbol demonstrate the pooled proportion and its 95%-CI.

For the implant failure rate, most studies observed few failures, with individual failure rates ranging from 0% to 50%. The common-effect model yielded a pooled failure proportion of 0.137 (95% CI 0.087–0.210, Figure 10). Between-study heterogeneity was negligible (*I*^2^ = 0%, τ^2^ = 0, *p* = 0.47), and the random-effects model therefore provided an identical estimate. Similarly, the funnel plot (Figure S5) did not reveal an obvious asymmetry, and Egger’s test was not feasible in this case. Leave-one-out influence analysis showed a pooled estimate remaining within the 95% CI of the primary model. Subgroup analysis was not feasible as well due to insufficient data. As there was only one study that reported radiotherapy after implantation and included implant survival/success, the meta-analysis was not feasible for the radiotherapy timing.

Overall, the certainty in the evidence was limited (Table S1). Studies were judged to have low certainty because of observational designs, substantial heterogeneity, and suspected publication bias. For implant success, complication rate, radiotherapy-related failure, the comparative effect of radiotherapy versus no radiotherapy, and secondary implant failure, the certainty of evidence was rated as low to very low owing to high risk of bias, sparse and imprecise data, heterogeneous outcome definitions and uncertain reporting bias. The pooled estimates for these outcomes should therefore be interpreted as indicative rather than definitive.

### 3.5. Risk of Bias and Quality Assessment of Included Studies

The ROBINS-E outcome for 14 retrospective studies is given in Table S2, with one reporting as very high, one reporting as some concerns and the remaining reporting with high risk of bias. Two cohort studies were assessed with the NOS scale (Table S3), with one demonstrating moderate risk of bias and the other with a low risk of bias.

## 4. Discussion

Although prognostic evaluation is critical, it is complex given the compromised condition and inconsistent treatment plans reported in studies [44]. According to the meta-analyses, implants placed in reconstructed jaws show a high survival rate and an acceptable success rate at a median follow-up of 44.5 months but at the cost of considerable uncertainty, with moderate to substantial heterogeneity and evidence of small-study effects. The situation is similar to the complication rate. For irradiated cases, the pooled failure rate was higher, with a high odds ratio, but the confidence intervals were wide and the certainty low. Leave-one-out and funnel plot analyses indicated that the results were robust to the exclusion of individual studies but vulnerable to publication and reporting bias. Taken together, the feasibility of implant rehabilitation after maxillomandibular reconstruction was supported, while underlining the need for better-designed, larger prospective studies to clarify long-term prognosis. In addition, based on clinical experience and the review, we advise considering the following aspects and cautiously making a treatment plan and prognosis evaluation on a personalized scenario.

### 4.1. Implant Survival and Success

In this systematic review, the pooled implant survival rate reached 97.9%, with a success rate of 88.1% during a follow-up period of at least 36 months. The pooled survival rate was similar to previous publications, with a mid-term result at 93.5% [1] and 93.2% [11], respectively. However, in a longer follow-up of 20 years, for instance, the survival rate in free bone flap reconstructed bones dropped to 69% [1]. Compared with implant survival, implant success was inconsistent across studies because success criteria varied [40]. A previous review mentioned the importance of success criteria; however, survival and success rate were not separated [25]. In the current review, included studies comprehensively defined the success criteria except Chang et al. [30], where success was presented as no bone loss, and Cuéllar et al. [31], where the success criteria were not given. Considering long-term clinical prognosis and living standards, application of specific success criteria is of critical importance [40].

Various factors from reconstruction to implantation and to prosthesis loading impact implant survival and success. For instance, the jaw-in-a-day technique has been discussed, as well as the performance of immediate implant in reconstructed jaws [45,46,47], as timing is crucial to patients’ quality of life regarding oral function [48,49,50]. In accordance with the previous review [51], contributing studies did not report implant failure in immediate implantation. However, considering bias in these small sample studies, this similarity is recommended for reference only. Additionally, owing to extreme conditions in patients receiving jaw reconstruction, such as malignant tumors, it is difficult to draw a consensus on the exact implant placement timing for these patients [25,52].

For patients undergoing maxillomandibular reconstruction due to malignant tumors, radiotherapy serves as a common adjuvant treatment modality [53]. Studies have reported with evidence that radiation may negatively influence blood supply, leading to fibrosis [31,53], affecting bone height and introducing bone resorption after fibula flap reconstruction [31], although other series have not found a statistically significant impact of radiotherapy on the survival of implants [8,32,54]. Additionally, a dose–response relationship has been described [53], and implant failure was observed to be more common in patients who underwent radiation therapy [31,35,38]. In the current review, the pooled failure rate of implants that underwent radiotherapy was 13.7%. The failure rate is considerably higher [55] compared to previous reports. The failure here was defined as implant loss or “sleep”, indicating that implants were nonfunctional. On the basis of these observational data, a plausible but unproven mechanistic explanation is that radiotherapy-induced vascular compromise and fibrosis may predispose patients to chronic inflammation and disturb bone remodeling, thereby increasing the risk of marginal bone loss and functional overload of implants.

There is no consensus on the recommended timing of radiotherapy, although in this review, more cases received radiotherapy before implantation. For pre-implant radiotherapy, it is advised that implantation should be delayed in order to avoid possible osteoradionecrosis. Supportively, radiation may mainly negatively affect early osseointegration as the risk of radiation decreased during follow-up [38]. The time mentioned in the included studies varied from 12 months to at least 24 months [8,31,32,35,37,38,40]. In other reported studies, Li et al. preferred post-implantation radiotherapy to reduce complications and improve initial osseointegration [56], supported by a higher implant survival rate in postoperative radiotherapy [51]. Beyond those observations, it has been hypothesized that a long interval after radiotherapy may lower the potential for recovery as progressive endarteritis increases with time [35]. To summarize, it may be worth managing a dentition-target surgery before maxillomandibular reconstruction to avoid excessively delayed implantation and to plan for suitable radiotherapy timing [35].

### 4.2. Peri-Implant Prognosis

According to the 2017 World Workshop on Periodontology, the diagnosis of peri-implantitis is defined as follows: presence of BOP/suppuration plus progressive bone loss beyond initial remodeling (or, without prior records, PD ≥ 6 mm and bone level ≥3 mm apical to the most coronal intra-osseous part) [57]. Also, peri-implant health is characterized by the absence of erythema, bleeding on probing, swelling and suppuration, and probing is a critical assessment of peri-implant health [57].

This systematic review paid attention to peri-implant outcomes, including marginal bone loss, PD, and bleeding index. To give a systematic overview of soft tissue condition, the included studies documented the follow-up with the index related to bleeding on probing [33]. There was an observed increasing trend of mSBI and PD during follow-up [41,42], and implants that were lost in free bone flaps had significantly higher mSBI and PD compared with the remaining implants [42]. PD also tended to be deeper around implants in vascularized free bone flaps, which is likely related to thicker soft tissue coverage and the presence of pseudo-pockets of 4–6 mm [33]. Moreover, there is currently no universally accepted PD cut-off that defines peri-implant health or disease [57]. Taken together, these findings suggest that PD is associated with the local inflammatory burden, but in reconstructed jaws, increased PD may also reflect anatomical features rather than true pathology. Therefore, for diagnosing peri-implantitis in this setting, greater emphasis should be placed on longitudinal changes in PD combined with other clinical signs of inflammation, rather than relying on a single PD threshold at one time point. Although the BOP criteria were inconsistent across studies, a prevalence of sulcus bleeding was documented in the included studies. BOP is the hallmark of mucositis, but alone it is an imperfect predictor of peri-implantitis [58], with only one-third of the BOP-positive spots showing peri-implantitis as implant sites that are prone to exhibiting bleeding [59]. Additionally, smoking patients with poor circulation may lack the sign of BOP [58]. The uncertainty and high false positive rate make both probing depth and bleeding on probing unsuitable signs for further treatment, and clinical signs of inflammation, as well as changes compared to baseline records, may be of more importance [57,60]. Based on current evidence and our observations, deeper probing depths in flap tissue may be considered an expected anatomical finding in reconstructed jaws, rather than necessarily indicative of pathology or peri-implantitis. In addition, BOP itself is not a reliable predictor of peri-implantitis. Therefore, these parameters should not be used as the sole criterion for diagnosing peri-implantitis in these cases. Instead, careful monitoring of peri-implant parameters over time is essential for accurate assessment.

Radiological follow-up of marginal bone loss is critical, as radiographic examination is currently the only diagnostic tool that can verify changes in bone level, and MBL remains the key parameter for distinguishing peri-mucositis from peri-implantitis [61]. However, the diagnostic MBL threshold for peri-implantitis is inconsistent, and comparison with baseline values has been suggested as a more reliable monitoring approach [62]. In the available literature, most studies used panoramic radiographs to assess MBL, with some applying peri-apical X-ray [39] or CBCT scans [30]. Periapical X-rays are commonly recommended for evaluating bone loss [58], yet they still suffer from limited standardization and reproducibility [63]. Panoramic images are more prone to distortion and magnification than periapical radiographs, although their accuracy can be improved to a comparable level with commercial software and appropriate calibration [38], typically by comparing the known implant length with its radiographic length before measuring MBL [42]. CBCT enables evaluation of both buccal and lingual walls but is associated with higher radiation dose [64] and metal artifacts [58,65,66]. Taken together, the heterogeneous use of imaging modalities, variable calibration procedures and inconsistent diagnostic thresholds across studies likely introduce substantial measurement variability in MBL, which partly explains why quantitative pooling was not feasible in this review and limits the strength of prognostic conclusions based on radiographic bone-level changes.

### 4.3. Complications

Peri-implantitis is an important complication, and the mentioned parameters are indicative of this condition [67]. Besides regular monitoring, patient characteristics could act as a hint to a higher risk of peri-implantitis [25], for example, cigarette usage, poor oral hygiene and cancer [14]. There is no consensus on a gold-standard surgical protocol or material for peri-implantitis treatment, and long-term outcomes remain unpredictable, while surgical treatment includes mechanical decontamination through open flap debridement (OFD), with or without reconstructive procedures like guided bone regeneration (GBR), depending on defect characteristics [68,69]. In the included studies, Wiesli et al. [14] and Ko et al. [35] mentioned the removal of implants affected by peri-implantitis, Ewers et al. [32] reported successful treatment, and Pellegrino et al. [38] reported partial survival.

Another highlighted complication in the current review is hyperplastic tissue growth. This soft tissue inflammation around the implant may be ascribed to pyogenic granuloma (PG) or peripheral giant cell granuloma (PGCG), often associated with local irritation and foreign material, which is accompanied by a high risk of bone loss and implant failure [38,70,71]. From the included study, Pellegrino et al. found that the presence of peri-implant hyperplastic tissue was correlated to the rate of marginal bone loss [38]. A possible reason for this tissue growth was the absence of firmly attached and keratinized mucosa on transplanted bone and prosthetic-related difficulties in managing oral hygiene [38,72]. In the included studies, management of peri-implant complications was described only in small case series and individual reports. Complete surgical excision of the lesion and debridement were generally considered essential, and explantation of the implant was sometimes performed in cases of repeated recurrence; adjunctive laser therapy has been reported as a possible additional option rather than a standard protocol [70]. Wang et al. described 11 cases with overgrowth of granulomatous tissue treated with Er/YAG hydro laser at the first- and second-year follow-ups, subsequently resorting to excision and palatal mucosa grafting after eight recurrences in 2015, with temporary stability [41]. In a later report, the same group again removed overgrown tissue with an Er/YAG hydro laser followed by individualized oral hygiene instructions, although three of seven cases still failed at the 3-year follow-up [42]. These interventions were not evaluated in controlled comparisons and were not analyzed quantitatively in the present review; therefore, they should be regarded as anecdotal observations from individual series rather than evidence-based treatment recommendations.

Soft tissue management is a critical aspect for peri-implant health [38,42,73,74]. However, there has been no consensus over the exact impact of soft tissue grafts to maintain peri-implant tissue health [75,76,77]. Palatal mucosa transplantation, thinning skin paddle and collagen matrix graft as ways for soft tissue management were mentioned in the included studies. Pellegrino et al. demonstrated that a palatal-mucosa grafted implant had a higher survival/success rate and a lower occurrence of peri-implantitis [38]. Unstable soft tissue is responsible for pocket formation and plaque accumulation, while keratinized mucosa improves anti-periimplantitis ability [35]. The thinning skin paddle technique, which is technically sensitive, did not change the characteristics of peri-implant soft tissue and fell short of the ultimate outcome [42], which was similar to collagen matrix [42]. As previously emphasized, soft tissue grafting alone would not necessarily lead to improved peri-implant conditions [78,79], as successful treatment requires an individualized and cautious procedure. Pre-surgical planning of reconstruction, including considerations of reconstructed bone height, prosthesis type, surgical technique and post-surgical hygiene is essential as each of these factors can significantly affect treatment outcomes [80].

This review has several limitations. First, due to the observational, small, retrospective design of the included studies, the pooled estimates are prone to bias. There was a lack of patient-level data and inability to adjust confounders such as smoking, diabetes, flap type and implant system. Additionally, the scales for risk of bias assessment could only be interpreted as an approximate evaluation. Second, the criteria and parameters used to define outcomes varied among different studies, especially regarding biological prognosis, which further complicated data synthesis. Third, heterogeneity was substantial in most syntheses. Although several subgroups have been explored, these factors did not sufficiently account for the observed outcomes. In conclusion, the certainty of evidence is low to very low for most outcomes, and the pooled estimates should be interpreted as indicative rather than definitive.

## 5. Conclusions

This systematic review provides an integrated overview of the mid-term prognosis of implants placed in jaws reconstructed with vascularized free bone flaps. Within the limitations of the underlying observational evidence, current data suggest that implant-related outcomes in these complex cases are generally acceptable, with high survival, moderately high success rates and overall stable peri-implant biological prognosis over follow-up. The risk of radiotherapy was also supported by the synthesized results. However, the evidence base is constrained by heterogeneous follow-up in many series, small sample sizes and the lack of standardized reporting on peri-implant tissue health and complications. Future studies should priorities well-designed prospective cohorts with longer observation periods, consistent definitions of survival, success and biological complications, standardized radiographic protocols and the routine inclusion of patient-reported functional and quality-of-life outcomes to better characterize long-term prognosis.

## Figures and Tables

**Table 1:** Summary of included studies and their cohort characteristics.

Study	Study Design	Patient No.	Average Age (Year)	Gender	Cause of Defect	Defect Location	Flap Type	Average Follow-Up (Month)	Start Time of Follow-Up
Kovács et al. 2000 [36]	RS	11	NR	6 F/5 M	Ame 4/SCC 5/Trauma 1/Ker 1	Mandible	Vascularized iliac crest grafts	43.6 (12–72)	Implantation
Ghana et al. 2004 [34]	RS	13	32 (17–50)	5 F/8 M	Ame 13	Mandible	Free fibula flap	40.1 (18–70)	Prosthesis Delivery
Iizuka et al. 2005 [4]	RS	28	NR	NR	Malignant tumor	Mandible	Free fibula flap	45	Implantation
Shaw et al. 2005 [8]	RS	81	58 (15–80)	32 F/49 M	SCC 65/Other tumor 16	Mandible and maxilla	Vascularized iliac crest grafts/Free fibula flap/Radius flap	42 (median, 3.6–168)	Implantation
Gbara et al. 2007 [33]	RS	30	60.8	12 F/18 M	Malignant tumor 18/Atrophy 10/Osteomyelitis 2	Mandible and maxilla	Free fibula flap	47	NR
Chang et al. 2014 [30]	PS	23	35.8 (20–65)	8 F/15 M	SCC 3/Ame 13/Osteoradionecrosis 4/Other tumor 2/Trauma 1	Mandible	Free fibula flap	58.3 (26–102)	Prosthesis Delivery
Shen et al. 2015 [39]	RS	10	31.6 (15–50)	5 F/5 M	Ame 10	Mandible	Free fibula flap	71.7 (59–83)	Prosthesis Delivery
Wang et al. 2015 [41]	RS	19	42.3 (28–55)	7 F/12 M	Ame 11/Ker 8	Mandible	Free fibula flap	42.5	Prosthesis Delivery
Sozzi et al. 2017 [40]	RS	22	48 (12–70)	10 F/12 M	Malignant tumor 10/Benign tumor 8/Atrophy 3/Trauma 1	Mandible and maxilla	Free fibula flap	93.6 (15.6–210)	Prosthesis Delivery
Kumar et al. 2018 [37]	PS	10	38.1	2 F/8 M	SCC 4/Trauma 3/Osteoblastoma 2/Osteoradionecrosis 1	Mandible and maxilla	Free fibula flap/iliac crest graft	42.7 (38–46)	Prosthesis Delivery
Pellegrino et al. 2018 [38]	RS	21	49.6	6 F/15 M	SCC 10/Ame 3/Other tumor 8	Mandible and maxilla	Free fibula flap	90.2 (20–204)	Implantation
Cuéllar et al. 2021 [31]	RS	24	51 (13–71)	9 F/15 M	SCC 13/Ame 6/Trauma 4/Agenesis 1	Mandible	Free fibula flap	44 (16–59)	NR
Wang et al. 2021 [42]	RS	24	47.5 (34–65)	8 F/16 M	Ame 15/Ker 4/SCC 3/Osteomyelitis 2	Mandible	Free fibula flap	36	Prosthesis Delivery
Wiesli et al. 2023 [14]	RS	17	53.8 (37–67)	7 F/10 M	Tumor 9/Atrophy 5/Trauma 2/Micrognathia 1	Mandible and maxilla	Free fibula flap	86.4 (2–12)	Ablative surgery
Ko et al. 2024 [35]	RS	18	45 (33–58)	2 F/16 M	SCC 15/Ame 3	Mandible	Free fibula flap	39 (6–60)	Prosthesis Delivery
Ewers et al. 2025 [32]	RS	37	50.7 (15.2–76.5)	18 F/19 M	Tumor 32/Trauma 4/Atrophy 1	Mandible and maxilla	Free fibula flap	54.2	NR

RS, retrospective study. PS, prospective study. F, female. M, male. Ame, ameloblastoma. Ker, keratocyst. SCC, squamous cell carcinoma. NR, not reported.

**Table 2:** Implant survival/success in patients with microvascular flaps.

Study	Implant No. in Bone Flap	Implant Timing (Implant No.)	Interval Time from Reconstruction to Implantation	Radiation Patient Timing and No. (Pre: Before Implantation; Post: After Implantation)	Interval Time Between Implantation and Radiotherapy	Failed Implant Rate and Implant No. with Radiotherapy	Soft Tissue Management (Received Implant No.)	Implant Survival Rate	Implant Success Rate	Whether Adapted Jaw-in-a-Day and Time for Prosthesis Delivery
Kovács et al. 2000 [36]	40	Secondary 40	6 months	NR	NR	NR	NR	97.60%	NR	NR
Ghana et al. 2004 [34]	43	Primary 43	-	NR	NR	NR	Palatal mucosa graft 43	100%	NR	NO, 7–12 months
Iizuka et al. 2005 [4]	37	Primary 37	-	Pre/Post/Both	NR	NR	NR	100%	NR	NR
Shaw et al. 2005 [8]	115	Secondary 115	12 months	Pre 38	12 months	NR	Debulking of flap tissue and free mucosal grafts from the hard palate/tuberosity 115	73.91%	NR	NO, NR
Gbara et al. 2007 [33]	121	NR	4–8 months, 5 for nonirradiated, 7 for irradiated	Pre 18	NR	NR	NR	96.60%	NR	NR
Chang et al. 2014 [30]	71	Primary 36/secondary 35	4–6 months after osteotomy for vertical distraction	NR	NR	0% (0/3)	Palatal mucosa graft 53	100%	NR	NO, 2 months after radiologically confirmed osteointegration
Shen et al. 2015 [39]	32	Primary 25/secondary 7	Until the fibula-mandible junction is achieved	NR	NR	NR	Palatal mucosa graft 32	100%	100%	NO, 9–15 months after implantation
Wang et al. 2015 [41]	51	Primary 4/secondary 47	8–12 months after reconstruction	No receiving	NA	NA	NR	100%	86.30%	NO, 3–5 months
Sozzi et al. 2017 [40]	92	Primary 3/secondary 19	7–24 months (mean 11 months)	Pre 5/Post 1	13–20 months before implantation	5.3% (1/19) (mixed pre- and post-data were provided)	Skin paddle removal preserving subcutaneous tissues	97.80%	NR	NO, 5–6 months
Kumar et al. 2018 [37]	34	Secondary 34	Implants were placed in patients following bony union of the fibula bone to the mandible.	Pre 4	At least 12 months before implantation	0% (0/13)	Subperiosteal dissection with denture-guided epithelial regeneration	100%	NR	No, 6 months with an interim denture, another 6–8 weeks of definitive
Pellegrino et al. 2018 [38]	108	Secondary 108	Mean 20.8 months (8–38 months)	Pre 7	At least the previous 2 years	16.5% (6/37)	Connective tissue 21/skin graft 20	86.50%	73.50%	NO, 4–6 months after implant placement, another ~6 months for definitive ones
Cuéllar et al. 2021 [31]	98	Secondary 98	6 months after iliac bone graft; 12 weeks after radiologically illustrating enough bone mass for vertical distraction; 4 months after double barrel fibula transplantation	Pre 10	1 year before implantation	14.6% (6/41)	NR	NR	93.90%	NO, 6 months after implantation (iliac); 4 months after implantation (distraction)
Wang et al. 2021 [42]	69	Secondary	NR	Post 2	NR	20% (1/5)	Collagen matrix 34/thinning skin paddle 35	NR	91.30%	NO, 9 months after implantation, 1 year for definitive ones
Wiesli et al. 2023 [14]	62	Previously implanted in the fibula	6–8 months before transplantation	No receiving	NR	NR	NR	83.90%	NR	No, provisional prosthesis placed during transplantation.
Ko et al. 2024 [35]	46	Secondary 46	The same time for interim denture	Pre 3	18 months before implantation	50% (3/6)	Palatal mucosal graft 46	NR	84.80%	No, 6–9 months for interim dentures in ameloblastoma patients, 12 months for cancer patients, and 18 months after radiotherapy; for definitive overdenture, 12–16 months, 16–20 months and 24–30 months post radiotherapy
Ewers et al. 2025 [32]	146	Primary 33/secondary 113	20.3 months ± 37.4	Pre 13/Post 10	More than 1 year after implantation; not mentioned in pre groups	NR	NR	86.90%	80.20%	No, 4–6 months after implantation

NR, not reported. NA, not applicable.

**Table 3:** Peri-implant parameters and related complications.

Study	Marginal Bone Loss (mm)(Mean/Scope)	Probing Depth (mm)	Peri-Implant Bleeding Status	Implant Related Complications	Total Complication Rate
Kovács et al. 2000 [36]	NR	NR	NR	Lack of osseointegration 1	2.40%
Ghana et al. 2004 [34]	<1.5	NR	NR	NR	NR
Iizuka et al. 2005 [4]	NR	NR	NR	NR	NR
Shaw et al. 2005 [8]	NR	NR	NR	Osteoradionecrosis 2	1.73%
Gbara et al. 2007 [33]	53.00% (<1)/29.91% (1–2)/17.09% (>3)	79.50% (2–3)/17.09% (4–6)/3.41% (>7)	Sulcus bleeding index, 1.05	Fail to osseointegrate 1/screw fracture 2/peri-implantitis 20/late failure 1	25%
Chang et al. 2014 [30]	Mesial 0.46, distal 0.53 (mean 0.50 ± 0.47)	NR	NR	Infection	NR
Shen et al. 2015 [39]	Mesial 0.27 ± 0.26, distal 0.33 ± 0.25 (mean 0.30 ± 0.25)	Mesial 3.09 ± 0.82, distal 3.33 ± 1.05, buccal 3.02 ± 1.13, lingual 3.23 ± 1.17 (mean 3.16 ± 1.06)	NR	NR	NR
Wang et al. 2015 [41]	0.46 y1/0.62 y2/0.70 y3 (mean 0.70 ± 0.62)	NR	Modified sulcus index, 0.6 y1/0.9 y2/1.0 y3 (mean 1)	Overgrowth of granulomatous tissue 11	21.60%
Sozzi et al. 2017 [40]	NR	NR	NR	Peri-implantitis 2/splitting of the cortical bone 1	4.30%
Kumar et al. 2018 [37]	NR	NR	NR	Peri-implantitis 2/Failure after loading 2	11.76%
Pellegrino et al. 2018 [38]	2.2 ± 1	3.80 ± 2.00	NR	Peri-implant mucositis 9.8% (y5) and 18.2% (y10)/peri-implantitis 8.7% (y5) and 15.8% (y10)/peri-implant hyperplasic tissue 14.8% (y5) and 20.3% (y10)	NR
Cuéllar et al. 2021 [31]	1.61 ± 0.46	NR	NR	NR	NR
Wang et al. 2021 [42]	1.23 y1/0.69 y2/0.32 y3 (mean 0.32)	2.89 y1/3.2 y2/3.4 y3 (mean 3.40 ± 0.57)	Modified sulcus index, 1.2 y1/2 y2/2.3 y3 (mean 2.3)	Overgrowth of granulomatous tissue 7	10.10%
Wiesli et al. 2023 [14]	23.1% (≤0.5)/4.8% (≥3.6)/Most (0.6–1.5)	3.2	Papillary bleeding index, 74% (0)/1% (3)	Peri-implantitis 10	16.10%
Ko et al. 2024 [35]	NR	NR	NR	Peri-implantitis 3	6.50%
Ewers et al. 2025 [32]	NR	NR	NR	Non-integrated 3/Peri-implantitis 10	9.60%

NR, not reported.

## Data Availability

No new data were created or analyzed in this study. Data sharing is not applicable to this article.

## Supplementary Materials

The following supporting information can be downloaded at: https://www.mdpi.com/article/10.3390/diagnostics16030435/s1, Table S1, Certainty of evidence; Table S2, ROBINS-E for retrospective study quality analysis; Table S3, NOS for prospective study quality analysis; Figure S1, Subgroup analysis for implant survival rate by (a) follow-up, (b) radiotherapy and (c) implant timing; Figure S2, Subgroup analysis for implant success rate by (a) radiotherapy and (b) implant timing; Figure S3, (a) Leave-one-out meta-analysis and (b) funnel plot for complication rate; Figure S4, (a) Leave-one-out meta-analysis and (b) funnel plot of the odds ratio of implant failure with radiotherapy; Figure S5, Forest plot of odds ratio of implant failure with radiotherapy on patient level. Figure S6, (a) Leave-one-out meta-analysis and (b) funnel plot of implant failure with radiotherapy. PRISMA_2020_checklist. Search strategy.
