# Supplementary material for: Mid-Term Outcomes, Biological Responses and Complications of Dental Implants in Maxillomandibular Reconstruction with Free Bone Flaps: A Systematic Review and Meta-Analysis
Source: Diagnostics (Basel). 2026 Feb 1;16(3):435. doi: 10.3390/diagnostics16030435 (PMC12896764; doi:10.3390/diagnostics16030435)
Supplement: Supplementary file 1 [file diagnostics-16-00435-s001.zip › Search strategy.pdf]

PubMed:

(((("free tissue flap*"[Title/Abstract] OR "free flap*"[Title/Abstract] OR "free tissue transfer flap*"[Title/Abstract] OR "microsurgical free flap*"[Title/Abstract] OR "Bone ((Transplantation"[Title/Abstract] OR "bone graft*"[Title/Abstract]) AND (("mandibular reconstructi*"[Title/Abstract]) OR ("maxillo mandibular reconstructi*"[Title/Abstract])) AND (("dental implant*"[Title/Abstract]) OR ("dental prosthesis implantation"[Title/Abstract]) OR ("surgical dental prosthe*"[Title/Abstract]))) OR ("Bone Transplantation"[MeSH Terms] OR "Free Tissue Flaps"[MeSH Terms]) AND "Mandibular Reconstruction"[MeSH Terms] AND ("Dental Implantation"[MeSH Terms] OR "Dental Implants"[MeSH Terms]))

Embase:

(‘tooth implant’/exp OR ‘tooth implantation’/exp) AND (‘mandible reconstruction’/exp OR ‘maxilla resection’/exp) AND (‘bone graft’/exp OR ‘bone transplantation’/exp OR ‘surgical flaps’/exp)

Scopus:

(‘tooth implant’ OR ‘tooth implantation’) AND (‘mandible reconstruction’ OR ‘maxilla resection’) AND (‘bone graft’ OR ‘bone transplantation’ OR ‘surgical flaps)
