# Supplementary material for: Mid-Term Outcomes, Biological Responses and Complications of Dental Implants in Maxillomandibular Reconstruction with Free Bone Flaps: A Systematic Review and Meta-Analysis
Source: Diagnostics (Basel). 2026 Feb 1;16(3):435. doi: 10.3390/diagnostics16030435 (PMC12896764; doi:10.3390/diagnostics16030435)
Supplement: Supplementary file 1 [file diagnostics-16-00435-s001.zip › Supplementary Table.pdf]

Table S1. Certainty of Evidence

Outcome	Starting level	RoB	Inconsistency	Indirectness	Imprecision	Publication bias	Final certainty
Implant survival	Low	Serious	Serious	Not serious	Not serious	Suspected	Very low
Implant success	Low	Serious	Serious	Not serious	Not serious	Suspected	Very low
Complication rate	Low	Serious	Serious	Serious	Not serious	Unclear	Very low
Failure rate in irradiated implants	Low	Serious	Not serious	Serious	Serious	Unclear	Very low
Radiotherapy vs no radiotherapy (OR)	Low	Serious	Serious	Serious	Serious	Unclear	Very low

Table S2 ROBINS-E for Retrospective Study Quality Analysis.

	Confounding	Measurement of Exposure	Selection of Participants	Post-exposure Interventions	Missing Data	Measurement of the Outcome	Selection of the Reported Result	Overall
Kovács et al.	High	Low	High	Some concerns	Some concerns	Some concerns	Some concerns	High
Ghana et al.	High	Low	High	Some concerns	Some concerns	Some concerns	Some concerns	High
Iizuka et al.	High	Low	High	Some concerns	Some concerns	Some concerns	Some concerns	High
Shaw et al.	High	Low	High	Some concerns	Some concerns	High	Some concerns	Very high
Gbara et al.	High	Low	High	Some concerns	Some concerns	Some concerns	Some concerns	High
Shen et al.	High	Low	High	Some concerns	Some concerns	Some concerns	Some concerns	High
Wang et al.	High	Low	High	Some concerns	Some concerns	Some concerns	Some concerns	High
Sozzi et al.	High	Low	High	Some concerns	Some concerns	Some concerns	Some concerns	High
Pellegrino et al.	High	Low	High	Some concerns	Some concerns	Some concerns	Some concerns	High
Cuéllar et al.	High	Low	High	Some concerns	Some concerns	Some concerns	Some concerns	High
Wang et al.	High	Some concerns	High	Some concerns	Some concerns	Some concerns	Some concerns	High
Wiesli et al.	High	Low	High	Some concerns	Some concerns	Low	Some concerns	High

Ko et al.	High	Low	High	Some concerns	Some concerns	Some concerns	Some concerns	High
Ewers et al.	Some concerns	Low	Some concerns	Some concerns	Some concerns	Some concerns	Some concerns	Some concerns

Table S3 NOS for Prospective Study Quality Analysis.

Study	Selection				Comparability	Outcome			
	Representativeness of the exposed cohort	Selection of the non-exposed cohort	Ascertainment of exposure	Demonstration that outcome of interest was not present at start of study	Comparability of cohorts on the basis of the design or analysis	Assessment of outcome	Was follow-up long enough for outcomes to occur	Adequacy of follow up of cohorts	Sum up
Chang et al.		1	1	1	1		1		5
Kumar et al.	1	1	1		2	1	1		7
