# Supplementary figures and images for: Mid-Term Outcomes, Biological Responses and Complications of Dental Implants in Maxillomandibular Reconstruction with Free Bone Flaps: A Systematic Review and Meta-Analysis
Source: Diagnostics (Basel). 2026 Feb 1;16(3):435. doi: 10.3390/diagnostics16030435 (PMC12896764; doi:10.3390/diagnostics16030435)

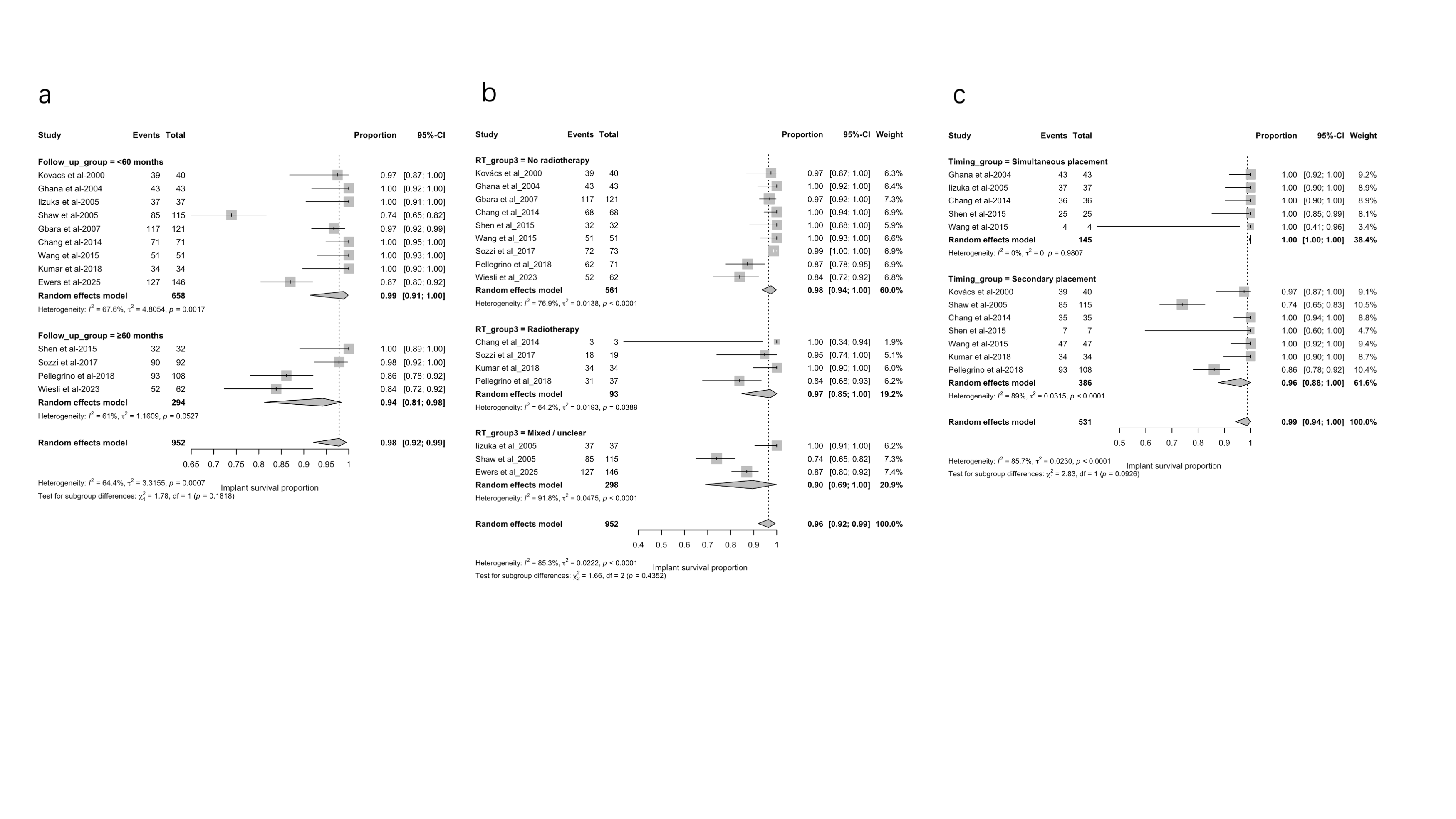

Supplement: Supplementary file 1 [file diagnostics-16-00435-s001.zip › Figure S1.tiff]

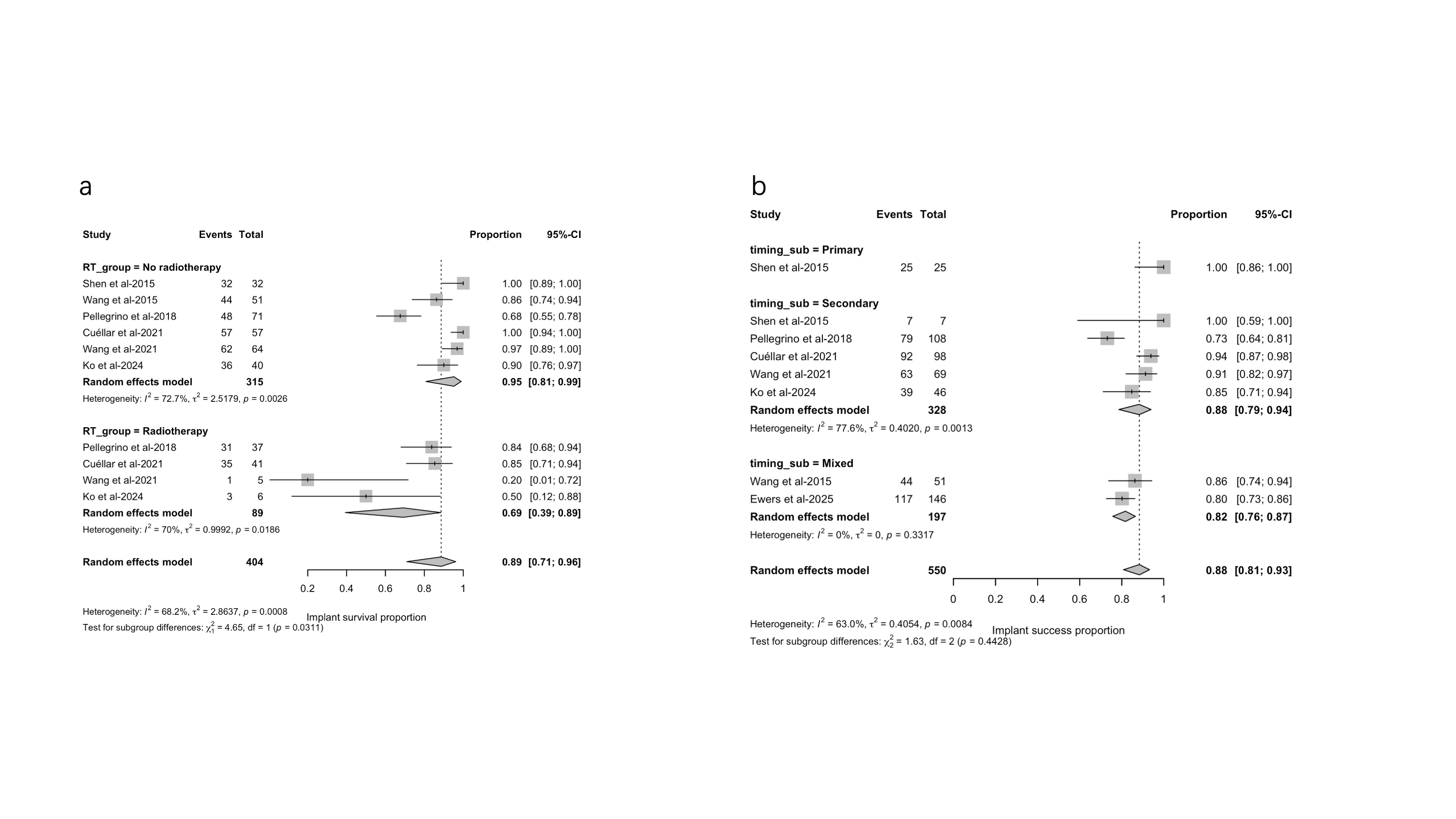

Supplement: Supplementary file 1 [file diagnostics-16-00435-s001.zip › Figure S2.tiff]

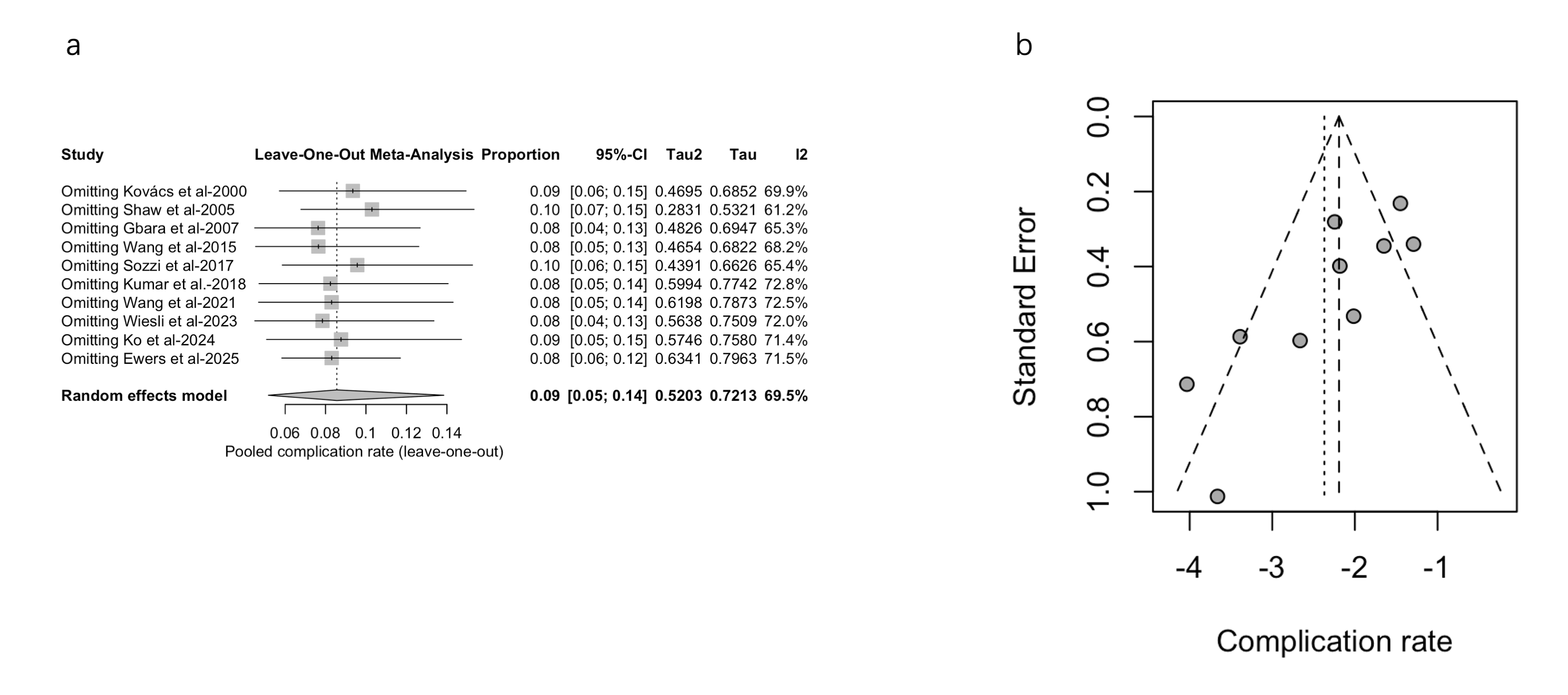

Supplement: Supplementary file 1 [file diagnostics-16-00435-s001.zip › Figure S3.tiff]

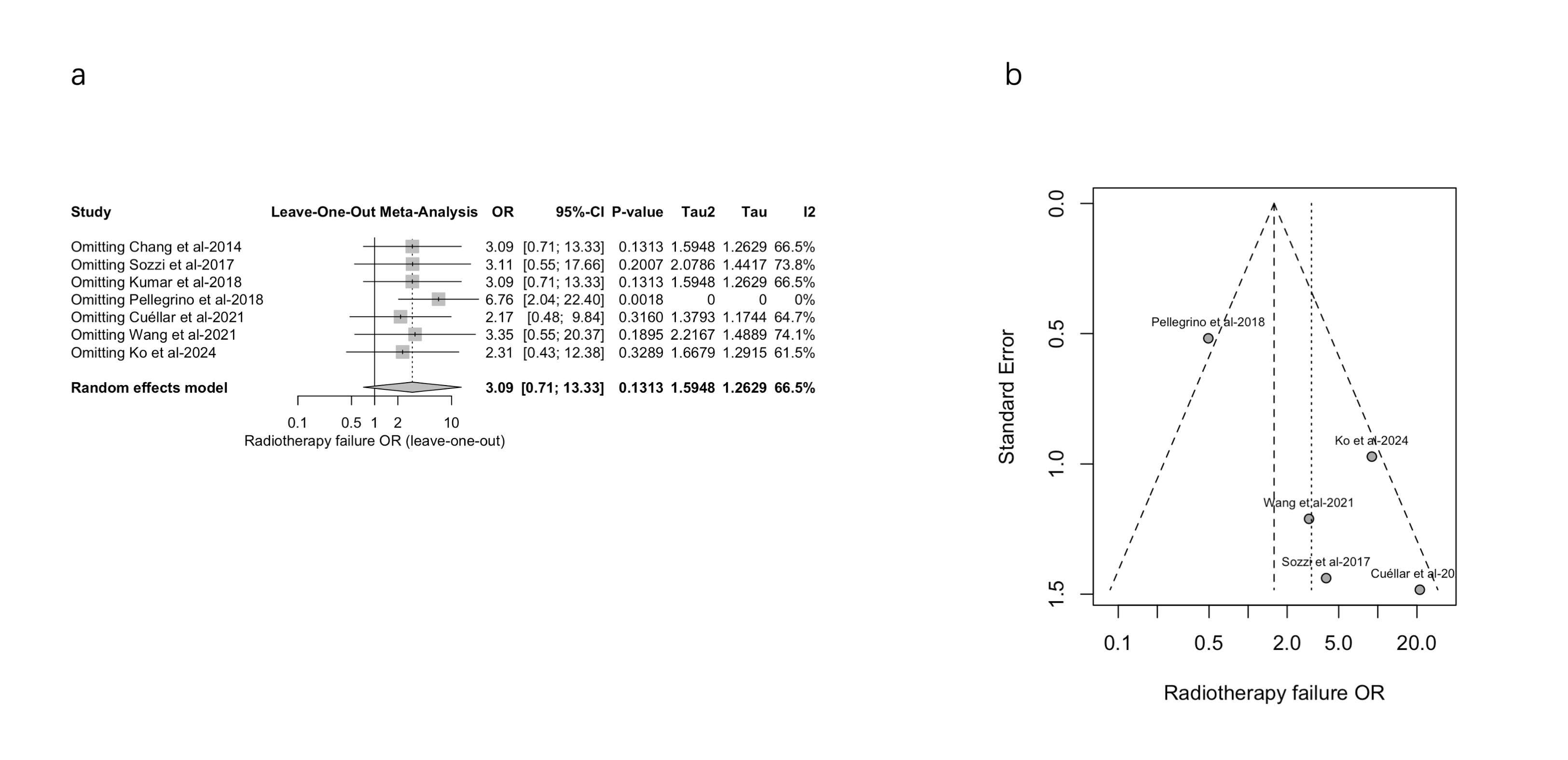

Supplement: Supplementary file 1 [file diagnostics-16-00435-s001.zip › Figure S4.tiff]

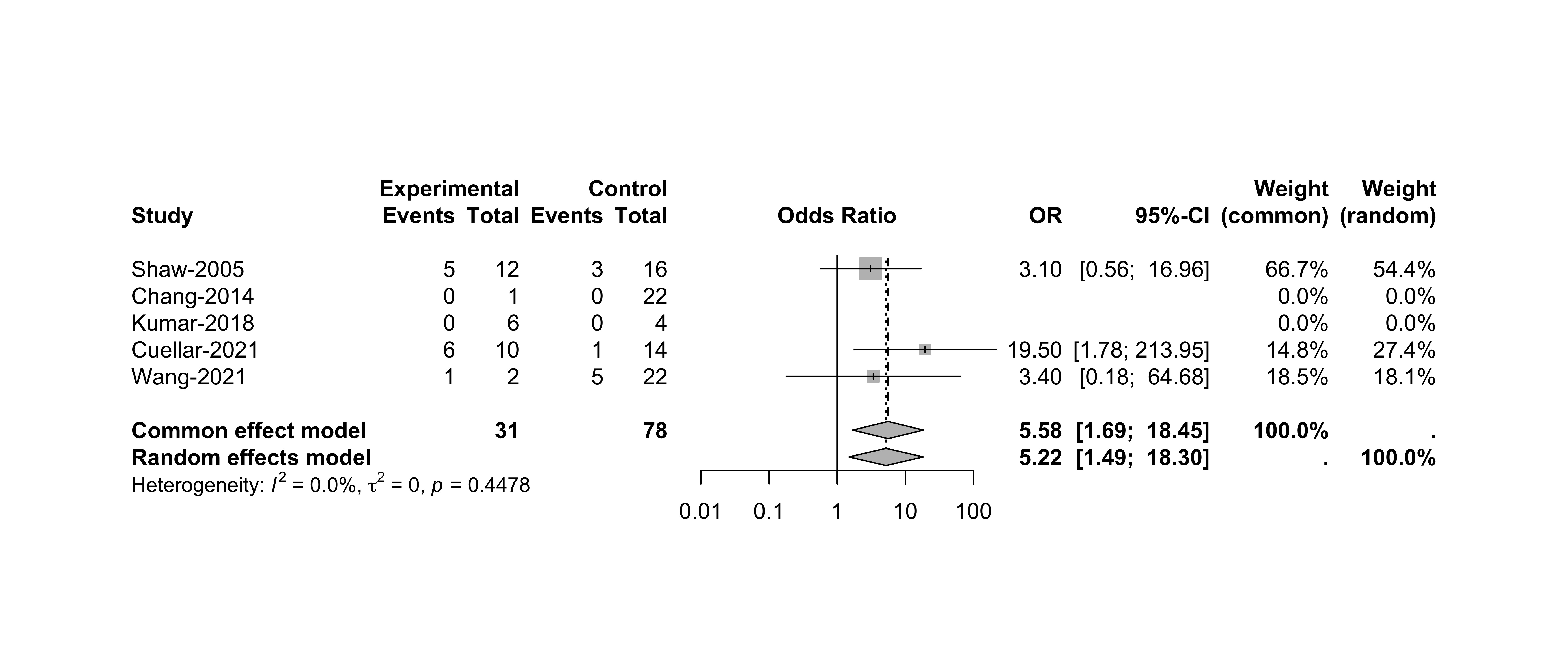

Supplement: Supplementary file 1 [file diagnostics-16-00435-s001.zip › Figure S5.tiff]

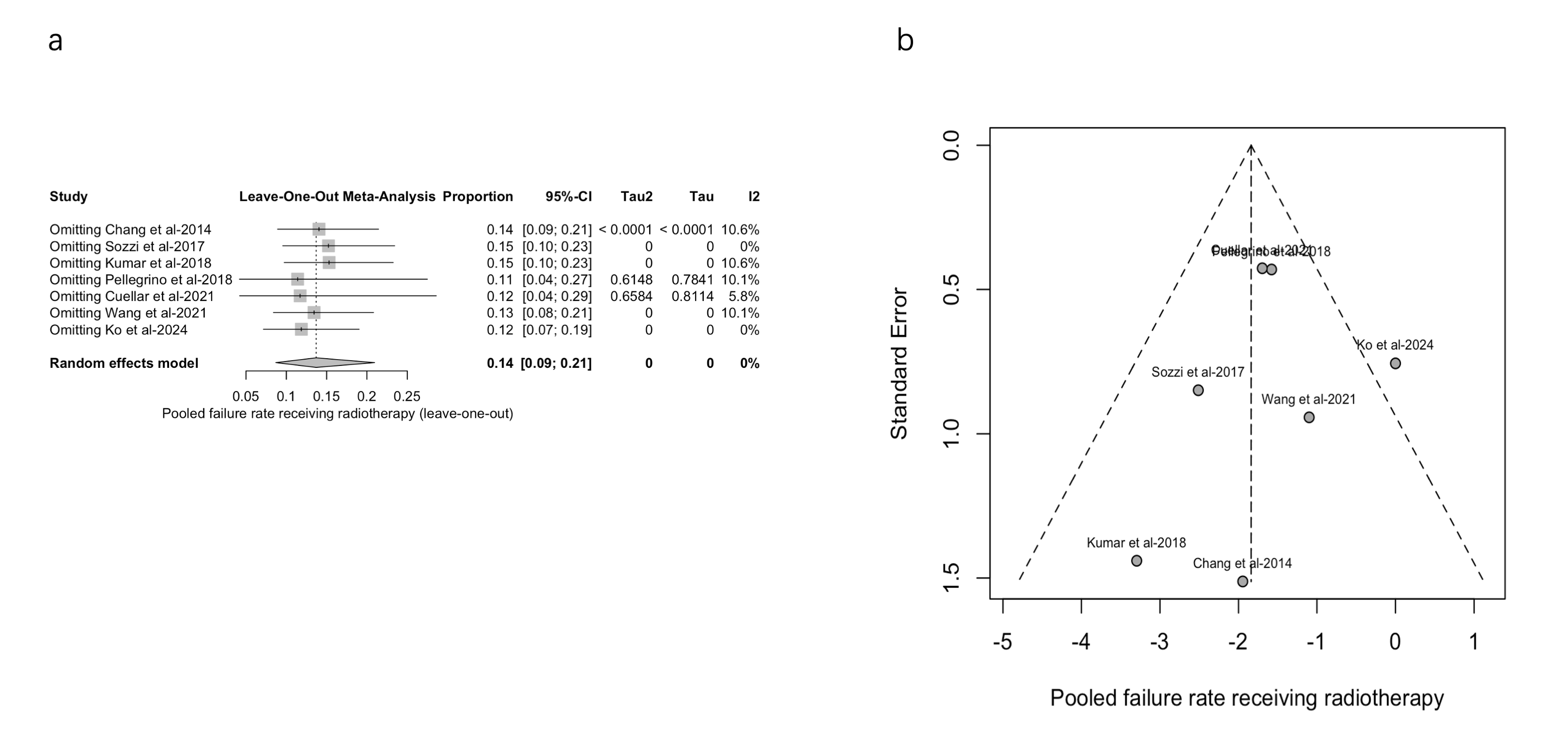

Supplement: Supplementary file 1 [file diagnostics-16-00435-s001.zip › Figure S6.tiff]
